# Surgical treatment of lung cancer with adjacent lobe invasion in relation to fissure integrity

**DOI:** 10.1111/1759-7714.13217

**Published:** 2019-12-18

**Authors:** Claudio Andreetti, Camilla Poggi, Mohsen Ibrahim, Antonio D'Andrilli, Giulio Maurizi, Matteo Tiracorrendo, Valentina Peritore, Erino Angelo Rendina, Federico Venuta, Marco Anile, Andreina Pagini, Giovanni Natale, Mario Santini, Alfonso Fiorelli

**Affiliations:** ^1^ Division of Thoracic Surgery, Sant'Andrea Hospital, Faculty of Medicine and Psychology University of Rome ‘Sapienza’ Rome Italy; ^2^ Division of Thoracic Surgery, Policlinico Umberto I, Faculty of Pharmacy and Medicine University of Rome ‘Sapienza’ Rome Italy; ^3^ Fondazione Eleonora Lorillard Spencer Cenci Rome Italy; ^4^ Division of Thoracic Surgery UniversitàdegliStudidella Campania “Luigi Vanvitelli” Naples Italy

**Keywords:** Adjacent lobe, fissure integrity, lung cancer, pleural invasion, surgery

## Abstract

**Background:**

Tumor with adjacent lobe invasion (T‐ALI) is an uncommon condition. Controversy still exists regarding the optimal resection of adjacent lobe invasion, and the prognostic value in relation to fissure integrity at the tumor invasion point. The aims of this paper were to evaluate the prognosis of T‐ALI with regard to fissure integrity, and type of resection.

**Methods:**

This was a retrospective multicenter study which included all consecutive patients with T‐ALI undergoing surgical treatment. Based on radiological, intraoperative and histological findings, T‐ALI patients were differentiated into two groups based on whether the fissure was complete (T‐ALI‐A group) or incomplete (T‐ALI‐D Group) at the level of tumor invasion point. Clinico‐pathological features and survival of two study groups were analyzed and compared.

**Results:**

Study population included 135 patients, of these 98 (72%) were included into T‐ALI‐A group, and 37 (38%) into T‐ALI‐D Group. T‐ALI‐D patients had better overall survival than T‐ALI‐A patients (63.9 ± 7.0 vs. 48.9 ± 3.9; respectively, *P* = 0.01) who presented with a higher incidence of lymph node involvement (35% vs. 4%; *P* = 0.004), and recurrence rate (43% vs. 16%; *P* = 0.01). At multivariable analysis, T‐ALI‐D (*P* = 0.01), pN0 stage (*P* = 0.0002), and pT≤5 cm (*P* = 0.0001) were favorable survival prognostic factors.

**Conclusions:**

T‐ALI‐D presented a better prognosis than T‐ALI‐A while extent of resection had no effect on survival. Thus, in patients with small T‐ALI‐D and without lymph node involvement, sublobar resection of adjacent lobe rather than lobectomy could be indicated.

**Key points:**

- The extent of resection of adjacent lobe had no effect on survival while T‐ALI‐D, pN0 stage, and pT≤5 cm were significant prognostic factors.
- In patients with small T‐ALI‐D and without lymph node involvement, sublobar resection of adjacent lobe could be indicated as an alternative to lobectomy.

## Introduction

Visceral pleural invasion (VPI) was adopted as a specific description in the Tumor, Node, Metastasis (TNM) classification of the International Union Against Cancer (IUAC) staging system in the mid‐1970s as T2, and this classification remained unchanged in the further revisions.^1^ Tumor with adjacent lobe invasion (T‐ALI) across the interlobar pleura was firstly defined as T2 in the sixth TNM classification of the Union for International Cancer Control (UICC), and in the seventh TNM classification proposed by the International Association for the Study of Lung Cancer (IASLC) it was classified as T2a unless other criteria assigned a high T category.^2^ The eighth edition of TNM classification revised the T category as follows: T2 tumor >5 cm and ≤7 cm are reclassified as T3, and T3 tumors >7 cm are reclassified as T4, but there has been no highlighted proposal for T‐ALI, and thus this should still be classified as T2.^3^ However, the TNM classifications proposed in subsequent years (including the last eighth edition by IALCS) do not consider the fissure status (complete or incomplete) in the definition of T‐ALI, and the data in the literature on this lack of classification is scarce and controversial.

The aims of our study were to: (i) Evaluate the prognosis of T‐ALI with regard to the fissure integrity; and (ii) define the most appropriate surgical resection for these patients.

## Methods

### Study design

This was a retrospective multicenter study which included all consecutive patients with T‐ALI undergoing surgical treatment in three different centers between January 2000 to January 2018. The data were extracted from the data base of each participating center. T‐ALI patients were differentiated into two groups based on whether the tumor invaded the adjacent lobe across a complete fissure point (T‐ALI‐A group), or directly invaded the adjacent lobe through an incomplete fissure point (T‐ALI‐D group). Exclusion criteria were: (i) Patients with T‐ALI simultaneously combined with invasion of chest wall, diaphragm, phrenic nerve, mediastinal pleura, and parietal pericardium. (ii) Patients with T‐ALI suspected on radiological and/or intraoperative findings but not confirmed by histological studies (ie. tumor that invaded only to the visceral pleura, and not to the adjacent lobe). (iii) Patients with a different histological diagnosis from NSCLC (ie. neuroendocrine, carcinoid, small cell carcinoma, oat cell carcinoma, sarcoma, and mesothelioma histology) as the natural history, treatment, and/or outcome of these histologic subtypes differ from those of NSCLC. (iv) Patients who received any kind of neoadjuvant treatment. (v) Patients with a history of concurrent malignant disease or other previous primary cancers.

The end points of the study were to evaluate the prognosis of T‐ALI in relation to the fissure integrity (primary end point), and to the extent of resection (secondary end point).

The study design was approved by local ethics committees of the coordinator center and approved by each participating center. All patients gave a written informed consent for the surgical treatment and were aware that all information could be used anonymously for scientific purposes only.

### Study population

Preoperative workup included total body computed tomography (CT). From 2008, PET scan has routinely been performed in all patients. Basic pulmonary functional test (PFTs) and blood gas analysis were routinely performed. Invasive staging of the mediastinum was performed, if indicated.

Resections included pneumonectomy, bilobectomy, and lobectomy associated with sublobar resection of the adjacent lobe (wedge resection or segmentectomy). The extent of resection was chosen by surgeon on the basis of patient pulmonary reserve and localization of the tumor. Complete lymph adenectomy was performed in all cases. All patients were restaged according to the eighth revision of the TNM proposed by IASLC.^3^ Patients with pathological advanced stage received adjuvant chemotherapy (CHT) or concurrent chemo‐radiotherapy (CHT/RT) according to standard clinical practice. T‐ALI were diagnosed by preoperative CT scan and intraoperative views and then confirmed by histological findings.

### Histological examination

All specimens were fixed with 10% formalin and embedded in paraffin. The tumors were cut in the horizontal section at 5 mm intervals to examine the fissure status and the ALI, and serial 4 μm sections were stained with hematoxylin and eosin. The Victoria blue van Gieson method was used to visualize the elastic fibers. In addition, the distance between the tumor and the parenchymal suture margin was also measured. Each pathological specimen was reviewed by two pathologists who were blinded to the clinical outcome of the study.

**Figure 1:**
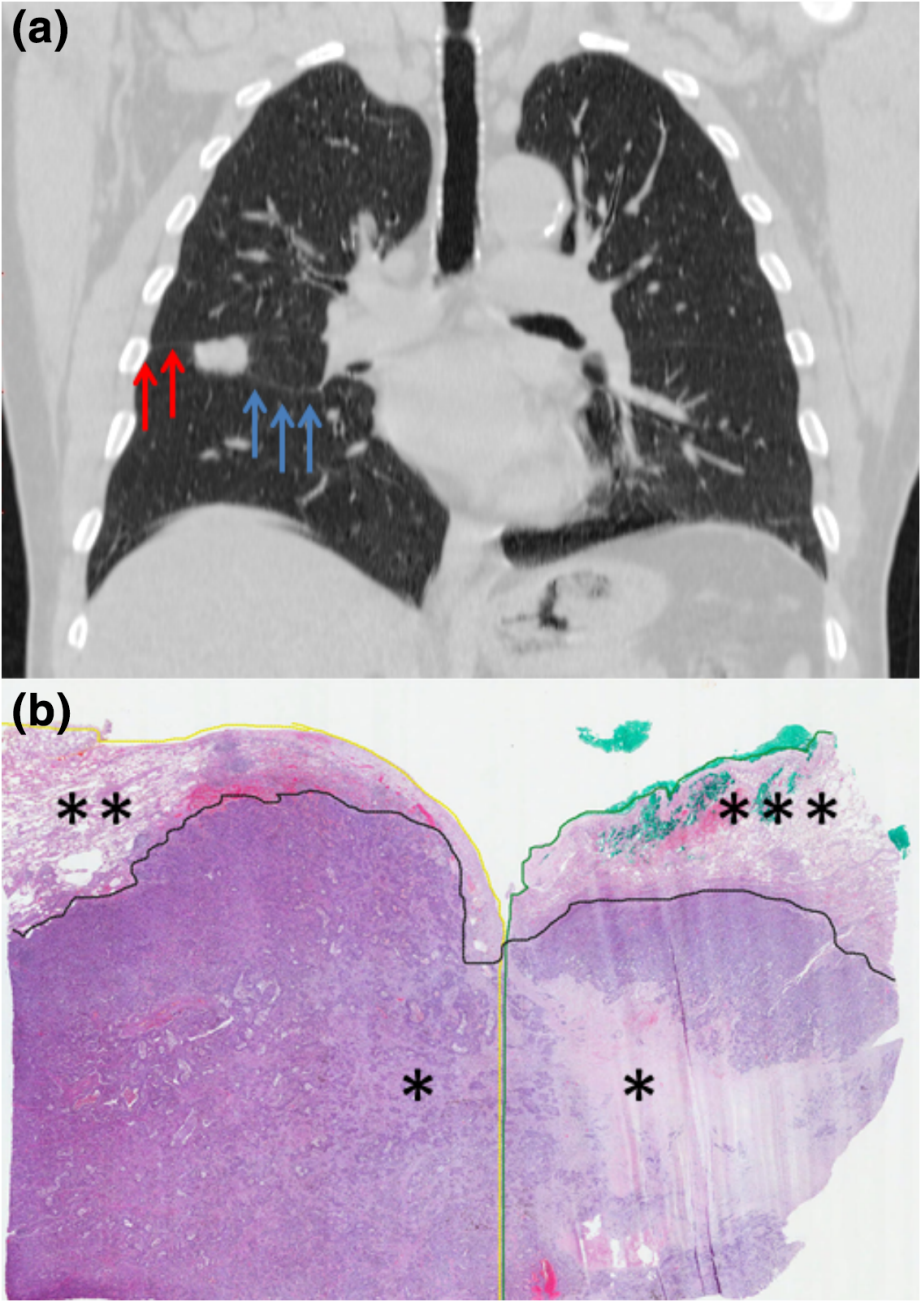
Radiological and pathological findings of T‐ALI‐D tumor. (**a**) Computed tomography scan of the chest showed adenocarcinoma of the middle lobe that invaded the apical segment (S6) of the lower lobe through a fissure that was fused in some areas (complete fissure: blue arrows; incomplete fissure: red arrows). (**b**) Pathological studies showed that tumor (*) of middle lobe (**) invaded S6 of the lower lobe (***) through an incomplete fissure point (black line: tumor; yellow line: middle lobe; green line: S6 of lower lobe; hematoxylin and eosin staining; 40× magnification).

**Figure 2:**
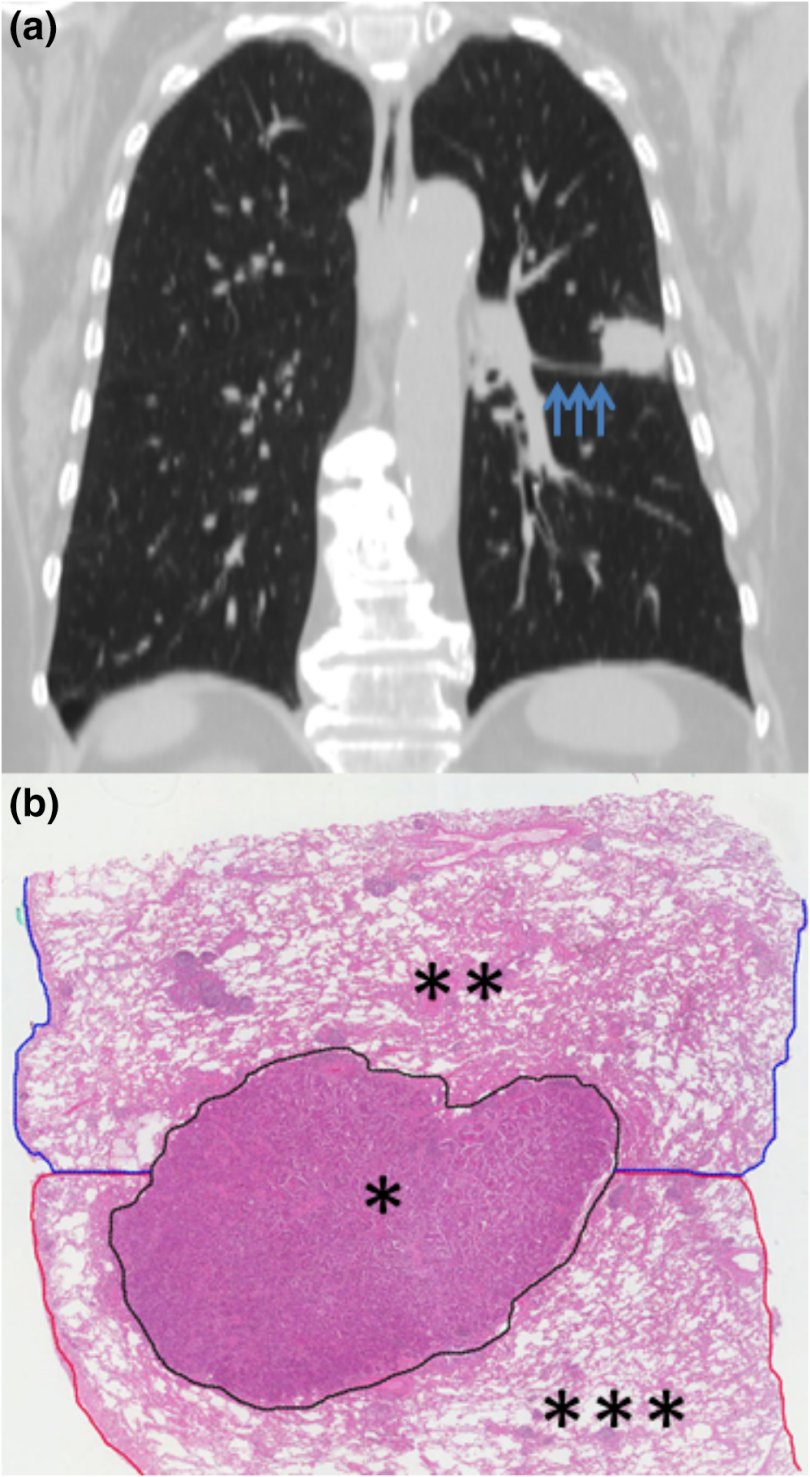
Radiological and pathological findings of T‐ALI‐A tumor. (**a**) Computed tomography scan of the chest showed squamous cell carcinoma of the left upper lobe that invaded the apical segment (S6) of the lower lobe across a complete fissure (blue arrows). The patient underwent upper lobectomy plus S6 segmentectomy of the lower lobe. (**b**) Pathological studies showed that tumor (*) of upper lobe (**) invaded S6 of lower lobe (***) through complete fissure point (black line: tumor; blue line: upper lobe; red line: S6 of lower lobe; hematoxylin and eosin staining; 40× magnification).

Example of T‐ALI‐D and T‐ALI‐A are reported in Figs 1 and 2, respectively.

### Follow‐up

A follow‐up examination was generally carried out every three months for the first two years, and six months thereafter. The examination included physical and radiological examination, either CT or PET, as indicated. Recurrences were defined by radiology and in some cases confirmed by pathology. As previously reported,^4^ loco‐regional recurrence was defined as any recurrence into ipsilateral chest wall/pleural, parenchymal, bronchial stump, hilar and/or mediastinal lymph node. Distal recurrences were defined as any recurrence not located in the ipsilateral thorax. Recurrences in patients who had simultaneous loco‐regional and distant recurrences were defined as distant recurrences. Patients with recurrence and/or distant metastasis underwent additional treatments according to standard clinical practice.

### Statistical analysis

The summary statistics of patients' characteristics were tabulated either as mean ± standard deviation (SD) for continuous variables or as number of patients and percentages for categorical variables. Student's *t*‐test and chi‐square test were used to compare different variables, as appropriate. Overall survival (OS) was defined as the interval between the time of surgery and date of death or censoring. OS was calculated with the kaplan‐meier method, and intergroup differences were evaluated by log‐rank test. Univariable analysis used the following variables: age (≤70 year‐old vs. >70 year‐old); gender (male vs. female); histology (adenocarcinoma vs. others); fissure integrity (T‐ALI‐A vs. T‐ALI‐D); type of resection (pneumonectomy/bilobectomy vs. lobectomy associated with sublobar resection), surgical margin of resection (<2 cm vs. ≥2 cm), lymph node involvement (pN0 vs. pN1/pN2). Variables having *P*‐value <0.05 at univariable analysis were included in multivariable analysis (Cox proportional hazard model), and prognostic factors were considered significant if *P*‐value was <0.05. MedCalc statistical software (Version 12.3, Broekstraat 52; 9030 Mariakerke; Belgium) was used.

## Results

In the study period, 2245 patients underwent surgical resection for lung cancer. Of these, 146 (6.5%) presented T‐ALI, but 11 were excluded from the analysis as three had chest wall invasion, five presented a histological diagnosis different from NSCLC (two SCLC, three atypical carcinoid), and three had concurrent malignant disease. Thus, our study population contained 135 patients summarized in Table 1.

**Table 1:** Study population

Variable	All	T‐ALI‐A	T‐ALI‐D	*P*‐value
Number of patients (%)	135	98 (72%)	37 (38%)	—
Age (year‐old)	68 ± 3,5	67 ± 1.8	68 ± 2.8	0.67
Sex (male)	97 (72%)	70 (71%)	27 (73%)	0.85
Type of resection				
Pneumonectomy	23 (17%)	18 (18%)	5 (13%)	0.27
Bilobectomy	42 (31%)	32 (33%)	10 (27%)	
Lobectomy + wedge	45 (33%)	35 (36%)	10 (27%)	
Lobectomy + segmentectomy	25 (18%)	13 (13%)	12 (33%)	
Histology				0.54
Squamous cell carcinoma	71 (53%)	50 (51%)	21 (56%)	
Adenocarcinoma	53 (39%)	40 (41%)	13 (35%)	
Large cell carcinoma	11 (8%)	8 (8%)	3 (9%)	
Main location + ALI				0.77
RUL + RML	47 (35%)	35 (36%)	12 (32%)	
RML + RLL	20 (15%)	13 (13%)	7 (19%)	
RUL + RLL	29 (21%)	21 (21%)	8 (22%)	
LUL + LLL	39 (29%)	29 (30%)	10 (27%)	
pTumor size	4.8 ± 1.3	4.7 ± 1.9	4.8 ± 1,1	0.49
pT1 (≤3 cm)	25 (18%)	19 (19%)	6 (16%)	
pT2 (>3 to 5 cm)	44 (33%)	32 (33%)	12 (32%)	
pT3 (>5 to 7 cm)	52 (39%)	38 (39%)	14 (39%)	
pT4 (>7 cm)	14 (10%)	9 (9%)	5 (13%)	
pN status				0.004
pN0	96 (71%)	63 (64%)	33 (90%)	
pN1	15 (11%)	13 (13%)	2 (5%)	
pN2	24 (18%)	22 (23%)	2 (5%)	
Surgical margin	26 ± 5.9	26 ± 1.3	26 ± 4.9	0.76
≤20 mm	12 (10%)	8 (8%)	4 (11%)	
>20 mm	123 (90%)	90 (92%)	33 (89%)	

ALI, adjacent lobe invasion; LLL, left lower lobe; LUL, left upper lobe; RLL, right lower lobe; RML, right middle lobe; RUL, right upper lobe.

The mean age of population was 68 ± 3.5‐year‐old with 72% being male patients. Pneumonectomy was performed in 23 patients (17%), bilobectomy in 42 (31%), and lobectomy with sublobar resection of the adjacent lobe in 70 patients, including 45 (33%) wedge resections, and 25 (18%) segmentectomies. Squamous cell carcinoma was observed in 71 patients (53%) and adenocarcinoma in 53 cases (39%). Stages pN0 included 96 (71%) patients undergoing pneumonectomy (*n* = 2; 2%); bilobectomy (*n* = 35; 36%); lobectomy plus segmentectomy (*n* = 20; 21%); lobectomy plus wedge resection (*n* = 39; 43%); Stage pN1 included 15 (11%) patients undergoing pneumonectomy (*n* = 10; 67%); bilobectomy (*n* = 2; 13%); lobectomy plus segmentectomy (*n* = 2;13%); lobectomy plus wedge resection (*n* = 1; 7%); and Stage pN2 included 24 (18%) patients undergoing pneumonectomy (*n* = 11; 46%); bilobectomy (*n* = 5; 21%); lobectomy plus segmentectomy (*n* = 3;12%); and lobectomy plus wedge resection (*n* = 5; 21%). The mean surgical margin was 2.6 ± 5.9 mm.

### Recurrence

Data are summarized in Table 2. The mean follow‐up was 38 ± 17 months (range: 6–83 months). There were a total of 48 recurrences in this study, representing 35% of the patient population. Loco‐regional recurrence was seen in 10 patients (7% of the entire population) undergoing pneumonectomy (*n* = 2); bilobectomy (*n* = 2), lobectomy plus segmentectomy (*n* = 3); and lobectomy plus wedge resection (*n* = 3; 30%). Pleura with malignant pleural effusion was the main site of local recurrence (six out of 10 cases, 60%), and five out of six (83%) cases were seen in T‐ALI‐A patients. No patients presented with recurrence in the resected surgical margin. Distant recurrences were found in 38 patients (28% of the population) undergoing pneumonectomy (*n* = 12); bilobectomy (*n* = 8), lobectomy plus segmentectomy (*n* = 8); and lobectomy plus wedge resection (*n* = 10). Among patients with distant recurrence, those with multiple locations were most common (11 out of 38; 29%). T‐ALI‐A was associated with a higher rate of recurrence than T‐ALI‐D (43% vs. 16%, *P* = 0.01); there was no significant difference in recurrence rate among patients undergoing bilobectomy or pneumonectomy and those undergoing wedge or segmentectomy (34% vs. 37%, *P* = 0.69).

**Table 2:** Characteristics of recurrence

Recurrence	All (*n* = 135)	T‐ALI‐A (*n* = 98)	T‐ALI‐D (*n* = 37)
Total	48 (35%)	42 (43%)	6 (16%)
Loco‐regional	10 (7%)	8 (8%)	2 (5%)
Lung	2 (1.5%)	2 (2%)	0
Pleura with malignant effusion	6 (4%)	5 (5%)	1 (2.5%)
Lymph node/mediastinum	2 (1.5%)	1 (1%)	1 (2.5%)
Distant	38 (28%)	34 (35%)	4 (11%)
Contralateral lung	4 (3%)	3 (3%)	1 (3%)
Contralateral chest wall	1 (2%)	1 (1%)	0
Brain	3 (2%)	2 (2%)	1 (3%)
Adrenal gland	4 (3%)	3 (3%)	1 (3%)
Liver	5 (4%)	4 (4%)	1 (3%)
Bone	3 (2%)	3 (3%)	0
Kidney	4 (3%)	4 (4%)	0
Multiple sites	3 (2%)	3 (3%)	0
Distant	11 (6%)	11 (12%)	0

Patients with recurrence underwent radiotherapy, chemotherapy, or combined chemotherapy and radiotherapy based on location of recurrence and their clinical condition. At the end of the time interval investigated, there were 37 deaths, while 10 patients were alive with NSCLC recurrence.

### Survival related to fissure status

**Figure 3:**
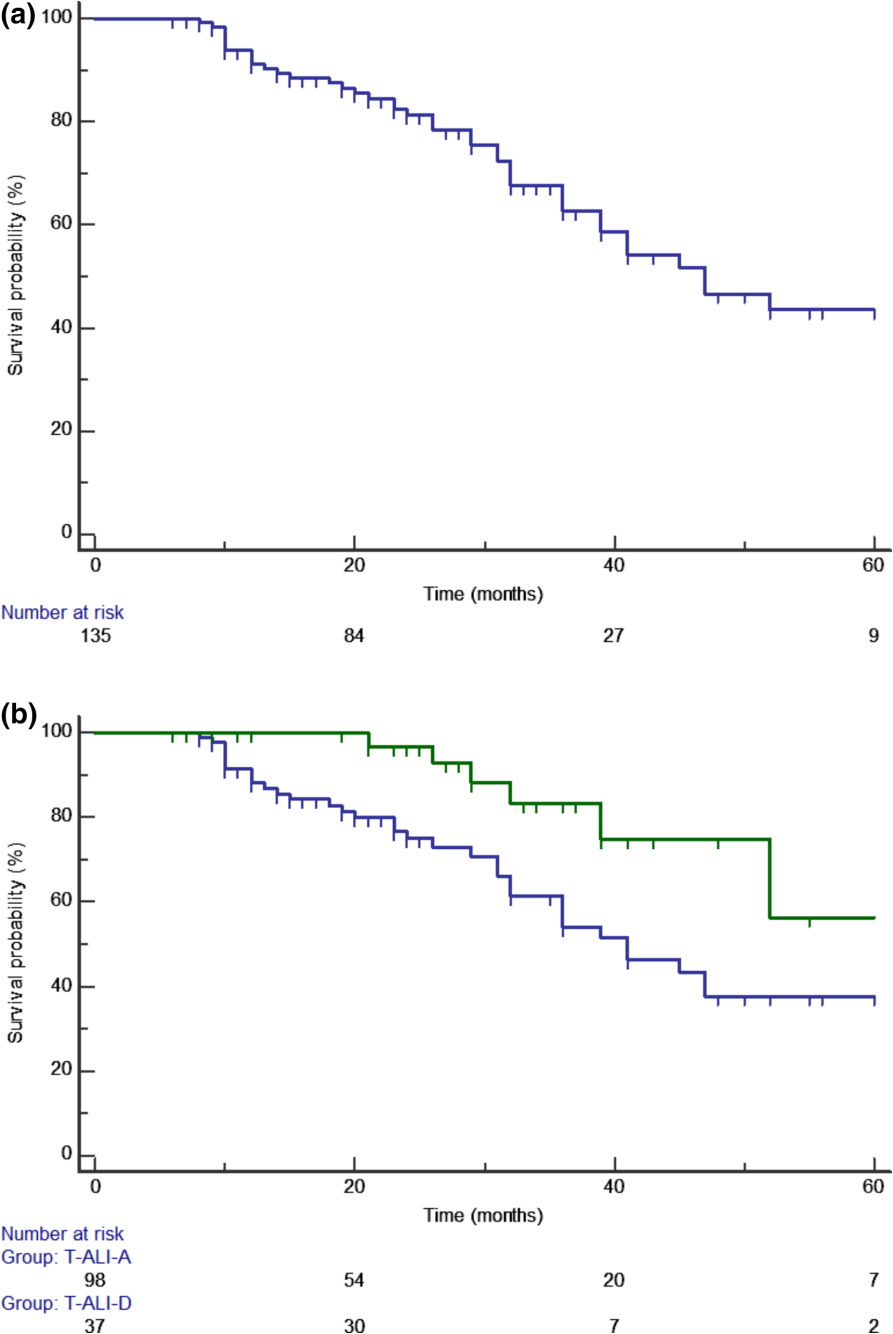
(**a**) The mean overall survival was 53 ± 3.3 months; (**b**) T‐ALI‐D showed a significant better survival than T‐ALI‐A (*P* = 0.01). (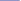) T‐ALI‐A, and (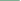) T‐ALI‐D.

The mean overall survival was 53 ± 3.3 months; three‐year survival rate (YSR) and five‐YSR were 62% and 43%, respectively (Fig 3a). Among all patient (*n* = 135), 98 (72%) were included into T‐ALI‐A group, and 37 (38%) into T‐ALI‐D group. As reported in Table 1, no significant intergroup differences were found regarding type of resection (*P* = 0.27), histology (*P* = 0.54), tumor location (*P* = 0.77), tumor size (*P* = 0.49) and suture margin (*P* = 0.76). T‐ALI‐A compared to T‐ALI‐D was associated with higher lymph node involvement rates (35% vs. 4%; *P* = 0.004), and higher recurrence rates (43% vs. 16%; *P* = 0.01). The survival of T‐ALI‐D patients was 63.9 ± 7.0 months; the three‐YSR, and five‐YSR were 62% and 43%, respectively. The survival of T‐ALI‐A patients was 48.9 ± 3.9 months; three‐YSR and five‐YSR were 54% and 37%, respectively. T‐ALI‐D showed a better survival than T‐ALI‐A (HR: 2.13; 95% CI:1.1–4.11; *P* = 0.01, Fig 3b).

### Survival related to extent of resection

**Figure 4:**
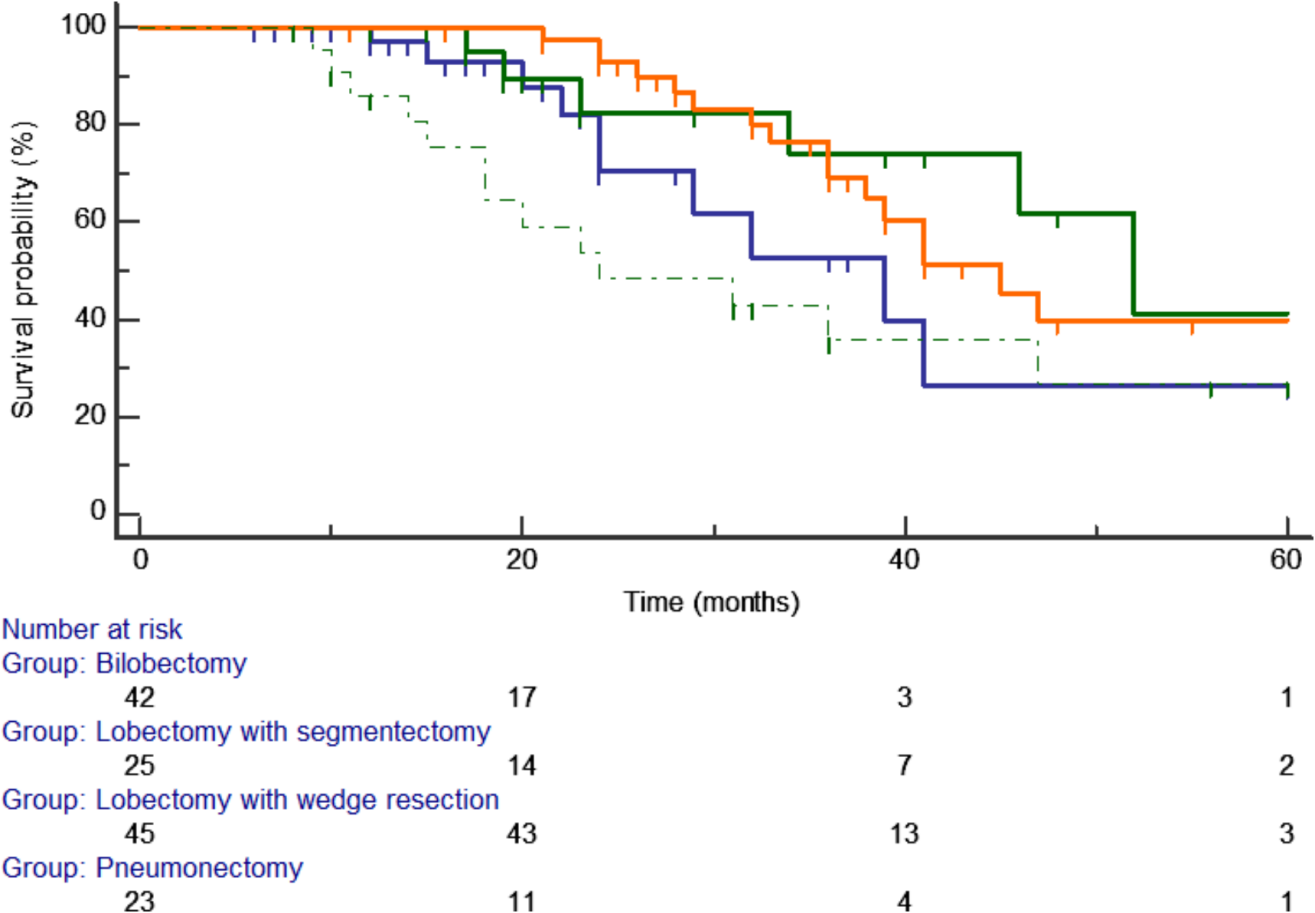
The mean overall survival of patients undergoing (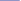) pneumonectomy, (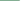) bilobectomy, (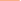) lobectomy with segmentectomy, and (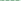) lobectomy with wedge resection was 39.8 ± 7.0; 53.9 ± 8.2; 54.3 ± 6.6; and 59.8 ± 4.8, respectively. Comparison of survival curves showed no significant difference (*P* = 0.09).

Survival of patients undergoing pneumonectomy, bilobectomy, lobectomy with segmentectomy, and lobectomy with wedge resection was 39.8 ± 7.0; 53.9 ± 8.2; 54.3 ± 6.6; and 59.8 ± 4.8 respectively (Fig 4). The comparison of survival showed no difference (*P* = 0.09). Lobectomy with sublobar resections group compared to pneumonectomy/bilobectomy group was associated with higher rates of pN0 patients (61% vs. 38%; *P* = 0.024) and of pT ≤5 cm patients (88% vs. 11%; *P* < 0.0001), while pneumonectomy/bilobectomy group was associated with higher rate of pN1/pN2 patients (72% vs. 28%; *P* = 0.006) and of pT >5 cm patients (13% vs. 87%; *P* < 0.0001). No significant difference regarding the rate of pneumonectomy/bilobectomy compared to that of lobectomy with sublobar resections was found in patients with a surgical margin ≤2 cm, and in those with a surgical margin >2 cm (Table 3).

**Table 3:** Type of resection in relation to pT stage and pN0 stage

		pT Stage (cm)				
pN Stage	Type of resection	≤3 (*n* = 25)	>3 to 5 (*n* = 44)	>5 to 7 (*n* = 52)	>7 (*n* = 14)	Total
pN0 (*n* = 96)	Pneumonectomy	—	—	1 (2%)	1 (7%)	2 (2%)
	Bilobectomy	1 (4%)	7 (16%)	21 (40%)	6 (43%)	35 (36%)
	Lobectomy + segmentectomy	12 (48%)	5 (12%)	3 (6%)		20 (21%)
	Lobectomy + wedge resection	5 (20%)	28 (64%)	6 (12%)	—	39 (43%)
pN1 (*n* = 15)	Pneumonectomy	—	—	7 (13%)	3 (21%)	10 (67%)
	Bilobectomy	—	—	2 (4%)	0	2 (13%)
	Lobectomy + segmentectomy	1 (4%)	1 (2%)	0	—	2 (13%)
	Lobectomy + wedge resection	1 (4%)	—	—	—	1 (7%)
pN2 (*n* = 24)	Pneumonectomy	—	—	7 (13%)	4 (29%)	11 (46%)
	Bilobectomy	—	—	5 (10%)	0	5 (21%)
	Lobectomy + segmentectomy	1 (4%)	2 (4%)	—	—	3 (12%)
	Lobectomy + wedge resection	4 (16%)	1 (2%)	—	—	5 (21%)

**Figure 5:**
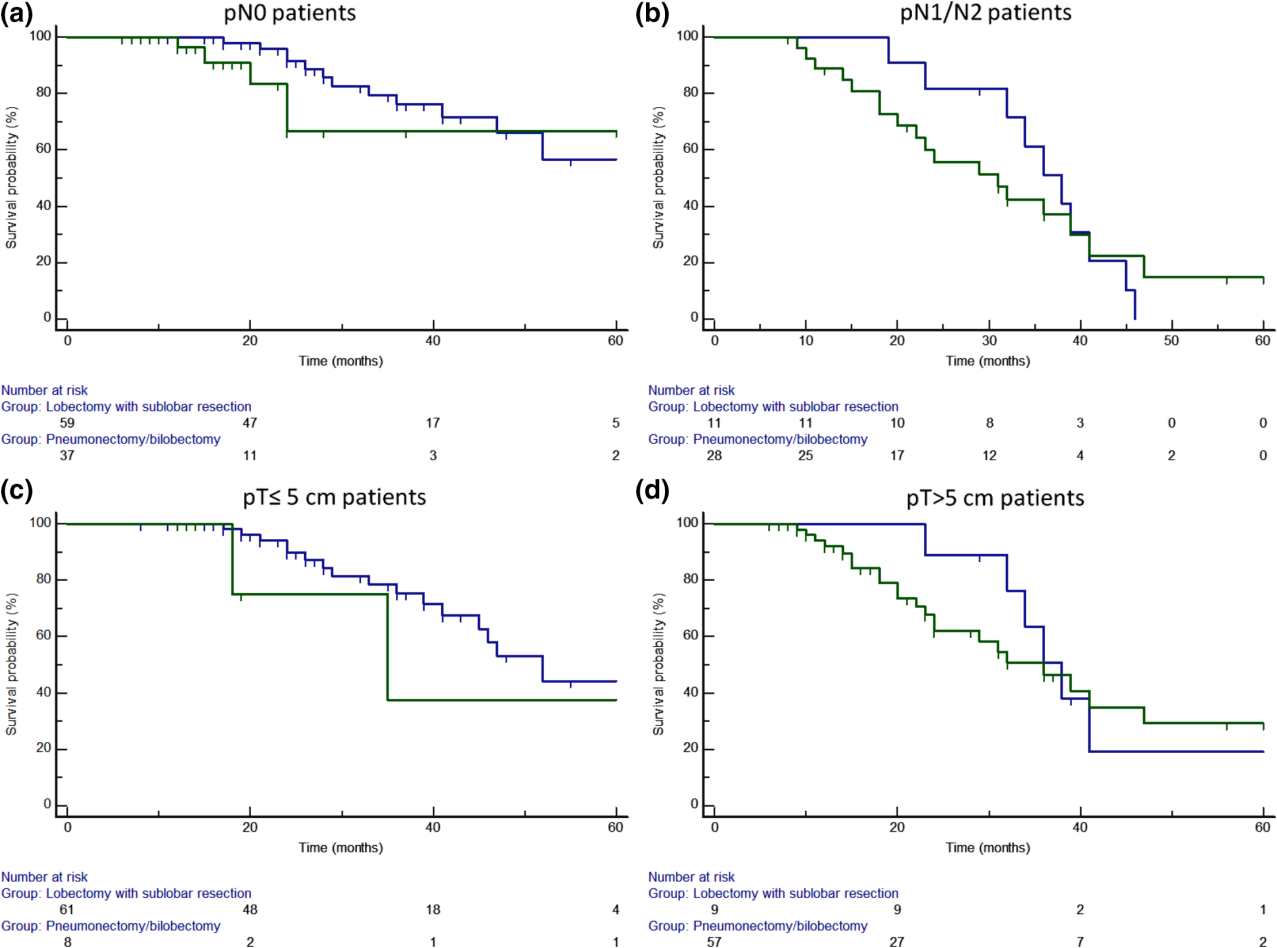
No significant difference was observed between patients undergoing (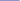) lobectomy with sublobar resections compared to those undergoing (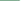) pneumonectomy/bilobectomy in relation to (**a**) pN0 status *(P* = 0.52); (**b**) pN1/pN2 status (*P* = 0.83); (**c**) pT ≤5 cm status (*P* = 0.40); and (**d**) pT > 5 cm (*P* = 0.93).

No significant survival difference was observed between patients undergoing lobectomy with sublobar resections compared to those undergoing pneumonectomy/bilobectomy in relation to pN0 (61.8 ± 4.7 month vs. 63.4 ± 8.2; *P* = 0.52; HR:0.6; 95% CI: 0.23–2.25; Fig 5a), to pN1/pN2 (31.8 ± 2.3 months vs. 35.6 ± 2.6, respectively; *P* = 0.83; HR:0.9; 95% CI: 0.23–2.25; Fig 5b), to pT ≤5 cm (47 ± 17 months vs. 53 ± 3.6 months, respectively; *P* = 0.40; HR:0.4; 95% CI: 0.06–2.98, Fig 5c), and to pT >5 cm (43.8 ± 5.3 months and 43.5 ± 7.9 months, respectively; *P* = 0.93; HR:0.96; 95% CI: 0.40–2.28; Fig 5d).

### Univariable and multivariable analysis for overall survival

The results are summarized in Table 4. On the univariable analysis T‐ALI‐D (HR:2.13; *P* = 0.01), pN0 stage (HR:3.75; *P* = 0.0004), pT ≤5 cm (HR:3.87; *P* = 0.0001), and margin resection >2 cm (HR:1.03; *P* = 0.04) were associated with a better survival. On the multivariable analysis T‐ALI‐D (HR:2.54; *P* = 0.01), pN0 stage (HR: 3.85; *P* = 0.0002), pT ≤5 cm (HR:3.91; *P* = 0.0001) were associated with a better survival.

**Table 4:** Cox regression analysis (dependent variable: overall survival)

	Univariable				Multivariable			
Covariates	Coefficient	HR	95% CI	*P*‐value	Coefficient	HR	95% CI	*P*‐value
**Age**								
≤70 vs. >70	0.42	1.52	0.52–1.34	0.19	—	—	—	—
**Gender**								
Male vs. female	0.46	1.19	0.89–1.78	0.65	—	—	—	—
**Resection**								
Pneumonectomy/bilobectomy vs. lobectomy with sublobar resection	−0.13	0.86	0.64–1.56	0.75	—	—	—	—
**Histology**								
Adenocarcinoma vs. others	0.76	0.45	0.75–1.87	0.69	—	—	—	—
**ALI**								
T‐ALI‐A vs. T‐ALI‐D	1.3	2.13	1.1–4.11	0.01	1.41	2.54	1.91–4.32	0.01
**pN stage**								
pN0 vs. pN1/pN2	1.29	3.75	1.59–3.45	0.0004	1.49	3.85	1.74–3.21	0.0002
**pT stage**								
***≤***5 cm vs. >5 cm	1.86	3.87	1.68–2.87	0.0001	1.86	3.91	1.68–2.87	0.0001
**Margin resection**								
≤2 cm vs. >2 cm	0.48	0.51	0.59–1.35	0.37				

CI, confidence interval at 95%; HR, hazards ratio.

## Discussion

T‐ALI is an uncommon condition with its incidence ranging from 5.5% to 17.1%.^5^ In this setting, prognosis is still debated; some authors^6^, ^7^ observed a survival rate similar to T2 NSCLC while others^8^, ^9^, ^10^, ^11^, ^12^, ^13^ reported a prognosis similar to T3 NSCLC. Controversy also exists concerning the type of resection; Yang *et al*.^11^ found that lobectomy of the adjacent lobe provided a better survival while other authors^7^, ^8^, ^9^ found that sublobar resection of the adjacent lobe was associated with a better survival. Different inclusion criteria, lymph node status and type of resection may explain these controversial results. T‐ALI was defined as T2 in the seventh TNM classification proposed by IASLC, and no further modification was considered in the eighth classification.^3^ However, previous TNM staging editions, including the eighth, do not consider the fissure status in the definition of T‐ALI, and only few reports observed T‐ALI outcome concerning this variable. Another limitation of previous studies was that T‐ALI was only suspected on the basis of CT images and operative findings, but then it was pathologically confirmed to be a simple interlobar fissure adhesion in most cases.

In our report, T‐ALI account for 6.8% of all tumors. In agreement with the findings of Nonaka *et al*.^6^ and Dziedzic *et al*.,^12^ the incidence of squamous cell carcinoma was higher than adenocarcinoma. A possible explanation is that squamous cell carcinomas are more often central tumors, and could preferentially be closer to the fissure or require pneumonectomy or broncho‐vascular sleeve resections.^14^, ^15^, ^16^

Most of the patients presented with a complete fissure (T‐ALI‐A), while only 30% had an incomplete one (T‐ALI‐D), especially on the right side. T‐ALI‐D had a better survival than T‐ALI‐A, in line with previous experiences. Ohtaki *et al*.^2^ found that T‐ALI‐A patients had a worse outcome than T‐ALI‐D (52% vs. 85.7%; *P* = 0.01) and therefore should be upstaged to T2b while T‐ALI‐D tumors had outcomes similar to T1 tumors. Similarly, Dziedzic *et al*.^12^ found a better three‐YSR in T‐ALI‐D patients than in T‐ALI‐A patients (67% vs. 58% *P* = 0.003). In theory, T‐ALI‐A presented with an aggressive biology as it invaded twice the pleural space with more chance of invading blood vessels and lymphatic ducts, of which the subpleural space is rich.^17^ Conversely, T‐ALI‐D grew from the primary to the adjacent lobe without invading visceral pleura and its lymphatic duct and blood vessels.^17^ Ohtaki *et al*.^2^ found that T‐ALI‐A compared to T‐ALI‐D presented a higher rate of vascular invasion (88% vs. 61%; *P* = 0.0099) and of lymphatic permeation (58% vs. 39%; *P* = 0.138). Yet, the study by Shimizu *et al*.^1^ reported that the invasion of the subpleural lymphatics resulted in a high rate of hilar and mediastinal lymph nodes involvement. In line with this evidence, T‐ALI‐A presented a higher rate of pN1 and pN2 involvement than T‐ALI‐D in our study. Similarly, Riquet *et al*.^18^ found pN1 in more than two‐thirds of T‐ALI patients since once the tumor invaded the adjacent lobe, it was able to drain in the lymphatic system of the adjacent lobe. Thus, the pN1 may be due to direct other lobe lymphatic invasion.

As lobectomy with systematic lymph node dissection is the current strategy for management of NSCLC in patients able to tolerate it,^19^, ^20^, ^21^ in theory T‐ALI should be treated as two primary tumors in different lobes. Thus, pneumonectomy, or at least bilobectomy (right lung) is more reasonable than lobectomy associated with sublobar resection in order to obtain a complete tumor resection and, thus reduce the risk of loco‐regional recurrence.^11^ Despite this evidence, in agreement with other authors,^7^, ^8^, ^9^ we found that pneumonectomy or bilobectomy provided no survival advantage compared to lobectomy associated with sublobar resection of an adjacent lobe. However, in our series, pneumonectomy or bilobectomy were performed at a higher rate in patients with tumor >5 cm and with pN1 and pN2 involvement, while in pN0 patients with tumor ≤3 cm lobectomy associated with sublobar resection was mainly performed. When the survival of different surgical resections was stratified with pN and with pT status, no significant difference was found, confirming the results of multivariable analysis that identified pN status, pT status as prognostic survival factors. Some authors found that a surgical margin ≥2 cm was associated with a lower incidence of recurrence after sublobar resections;^22^, ^23^ however, in our series this variable did not influence survival. Possible explanations are that only a small number of patients in our series (*n* = 12) had a surgical margin <2 cm, but, in all these, surgical margin was ≥1.5 cm and a R0 resection was obtained. Previously, our group found that surgical margin did not influence recurrence or the survival rate after wedge resection when an R0 resection was achieved.^24^ Yet, Mohiuddin *et al*.^25^ reported that in wedge resection for NSCLC, increasing the margin distance ≥15 mm significantly decreased the local recurrence risk, without evidence of additional benefit beyond 15 mm, while Wolf *et al*.^26^ found that a margin distance >11 mm was associated with longest overall survival. Confirming that, in our series, recurrence was not associated with the type of resection and no recurrences were found in the cut end of the lung parenchyma of the adjacent lobe. Similarly, Nonaka *et al*.^6^ and Leuzzi *et al*.^27^ showed that recurrence rate did not differ among patients undergoing bilobectomy or pneumonectomy and those undergoing wedge or segmentectomy of ALI.

Thus, if pneumonectomy or bilobectomy are the only procedure to obtain a complete resection of the tumor and lymph node involvement, these resections should not be rejected a priori only because they are associated with a higher risk of morbidity and mortality compared to more limited resection. Demir *et al*.^9^ (66.6%), Haam *et al*.^10^ (58.7%), and Riquet *et al*.^18^ (55.2%) reported a higher rate of pneumonectomy compared to other types of resection. Despite pneumonectomy having a lower five‐year survival rate when there was less extensive resection, these authors^10^, ^12^, ^18^ highlighted that the prognostic value of pneumonectomy was not related to the type of resection itself, but to the size of the tumor, the high rate of LN metastases, and/or its invasion beyond the visceral pleura, all factors that require extensive resection. Obviously, the choice of performing pneumonectomy, especially if on the right side, requires careful patient selection before surgery and intensive care after surgery and the decision should be made by the balance of obtaining a radical tumor resection, and preserving a good clinical status.^28^

Several limitations should be considered when interpreting our results. The main limitation is the retrospective nature of the study as the choice of resection was chosen by the surgeon's preference rather than randomization. The variations in surgical techniques and histological assessment due the multicenter nature are additional factors that may have affected our results. Yet, due to the small number of patients we included in the same group pneumonectomy and bilobectomy, despite the different extent of resection, neither propensity score match analysis was performed to limit the difference among different subgroups.

In conclusion, our data showed that prognosis of T‐ALI tumor was significantly associated with fissure integrity, while the extent of the resection did not affect patient survival. In theory, lobectomy with sublobar resections could be the preferred strategy in patients with small T‐ALI‐D and without lymph node involvement while more extensive resections could be indicated in advanced T‐ALI‐A in order to obtain a complete resection of the tumor and lymph nodes. Future prospective randomized studies should confirm our results.

## Disclosure

The authors declare no conflicts of interest.
